# Brain zinc chelation by diethyldithiocarbamate increased the behavioral and mitochondrial damages in zebrafish subjected to hypoxia

**DOI:** 10.1038/srep20279

**Published:** 2016-02-08

**Authors:** Marcos M. Braga, Emerson S. Silva, Tarsila B. Moraes, Gabriel Henrique Schirmbeck, Eduardo P. Rico, Charles B. Pinto, Denis B. Rosemberg, Carlos S. Dutra-Filho, Renato D. Dias, Diogo L. Oliveira, João Batista T. Rocha, Diogo O. Souza

**Affiliations:** 1 Programa de Pós-graduação em Bioquímica, Departamento de Bioquímica, Instituto de Ciências Básicas da Saúde, Universidade Federal do Rio Grande do Sul, 90035-003 Porto Alegre, RS, Brazil; 2 Universidade Federal do Pampa, Campus São Gabriel, 97.300-000 São Gabriel, RS, Brazil; 3 Instituto Nacional de Ciência e Tecnologia em Excitotoxicidade e Neuroproteção; 4 Programa de Pós-Graduação em Ciências da Saúde, Unidade Acadêmica de Ciências da Saúde, Universidade do Extremo Sul Catarinense, 88806-000, Criciúma, SC, Brazil; 5 Programa de Pós Graduação em Bioquímica Toxicológica, Departamento de Bioquímica e Biologia Molecular, Centro de Ciências Naturais e Exatas, Universidade Federal de Santa Maria, RS, 97105-900, Santa Maria, RS, Brazil; 6 Zebrafish Neuroscience Research Consortium (ZNRC), USA

## Abstract

The increase in brain levels of chelatable zinc (Zn) in dysfunctions involving oxygen deprivation has stimulated the treatment with Zn chelators, such as diethyldithiocarbamate (DEDTC). However, DEDTC is a redox-active compound and it should be better evaluated during hypoxia. We use the hypoxia model in zebrafish to evaluate DEDTC effects. The exploratory behavior, chelatable Zn content, activities of mitochondrial dehydrogenases, reactive species levels (nitric oxide, superoxide anion, hydroxyl radical scavenger capacity) and cellular antioxidants (sulfhydryl, superoxide dismutase) of zebrafish brain were assessed after recovery, with or without 0.2 mM DEDTC. The increased brain levels of chelatable Zn induced by hypoxia were mitigated by DEDTC. However, the novel tank task indicated that DEDTC did further enhance the exploratory deficit caused by hypoxia. Furthermore, these behavioral impairments caused by DEDTC were more associated with a negative action on mitochondrial activity and brain oxidative balance. Thus, due to apparent pro-oxidant action of DEDTC, our data do not support its use for neuroprotection in neuropathologies involving oxygen deprivation.

Zinc (Zn) is a metal abundantly found in the central nervous system (CNS)^1^. Despite the most part of Zn is usually bound to proteins, about 20% of total Zn is free or loosely bound to biomolecules in the brain^2^, both corresponding to the content of Zn susceptible to chelation. Chelatable Zn presents an important function to synaptic physiology, acting as neuromodulator on several molecular targets^3^. However, the accumulation of chelatable Zn in brain tissue may occur during ischemic stroke and hypoxic episodes, leading to oxidative stress and inflammatory response^4^,^5^. For this reason, the use of Zn chelators has been considered to mitigate the increased Zn levels during these brain dysfunctions^5^,^6^.

In particular, the diethyldithiocarbamate (DEDTC) is a chelator highly selective for Zn^7^ able to cross the blood-brain barrier^8^. Previous studies showed that DEDTC is beneficial against several disorders^9^,^10^. Due to Zn-binding properties, it has been suggested that DEDTC may play a neuroprotective role against hypoxia. However, the application of DEDTC as protective drug must be thoroughly investigated, since it is a redox-active compound that may impair cellular antioxidant/oxidant homeostasis^11^,^12^.

The use of a recent model of severe hypoxia in adult zebrafish^13^ emerges as a useful strategy to evaluate the mechanisms triggered by DEDTC on cerebral dysfunctions involving oxygen (O_2_) deprivation. As advantages, the use of zebrafish allows the researcher to investigate the effects of several drugs on large scale manner in a non-invasive model of hypoxic condition. Previous studies have shown that the hypoxia model in zebrafish causes brain damage similar to those triggered by hypoxia and ischemia in mammals^13^,^14^. Additionally, behavioral impairments and changes on brain mitochondrial function were also observed in fish 1 h after hypoxia^14^, representing a more refined protocol when compared to those described for rodents. The induction of hypoxia in zebrafish has been employed to evaluate the effects of DEDTC on the increased levels of chelatable Zn^15^. Thus, additional investigation using this model could straightforward the role of DEDTC on behavioral functions and oxidative status triggered by hypoxia.

Here, we evaluated whether the decrease in brain chelatable Zn performed by DEDTC improves the behavioral impairment and mitochondrial damage induced by hypoxia in zebrafish. Moreover, brain oxidative status afforded by DEDTC was investigated.

## Results

### Brain Chelatable Zn

**Figure 1:**
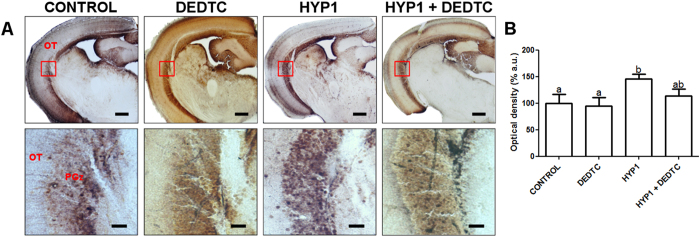
Chelatable Zn in the PGz of zebrafish brain stained by Neo-Timm after hypoxia followed by normoxic treatment, with or without DEDTC. (**A**) Chelatable Zn staining. Images in lower panels represent higher magnification of the area delimited by the square depicted in upper panels. The higher chelatable Zn content is indicated by the presence of the darker granules. (**B**) Optic densities of periventricular gray zone. OT, optic tectum; PGz, periventricular gray zone. Bars represent 200 μm in upper panels, and 60 μm in lower panels. In (**B**) the values are presented as the mean ± SEM (*n* = 3–4 per group). Different letters indicate statistical differences between groups at *p* < 0.05 level (two-way ANOVA followed by Tukey’s post hoc test).

Two-way ANOVA analysis showed a significant effect of hypoxia (*F*[3, 9] = 16.723; *p* = 0.003) and DEDTC (*F*[3, 9] = 5.281; *p* = 0.046) on the levels of chelatable Zn in brain tissue (*n* = 3–4 per group). The histochemical staining (Fig. 1A) and the optical density quantification of the periventricular gray zone (PGz) (Fig. 1B) demonstrated that hypoxia significantly increased chelatable Zn content in PGz (HYP1). However, DEDTC treatment attenuated the enhancement of chelatable Zn levels induced by hypoxia (HYP1 + DEDTC group). Moreover, DEDTC alone did not promote significant changes on chelatable Zn content.

### Behavioral Activity

**Figure 2:**
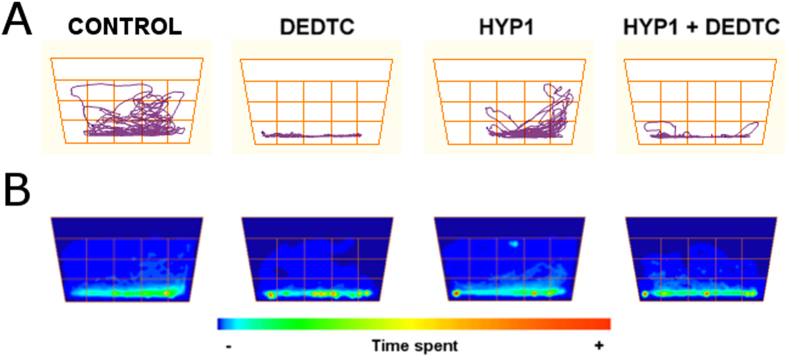
Exploratory profile of zebrafish after hypoxia followed by treatment, with or without DEDTC. (**A**) The representative track plots illustrating the swimming traces in apparatus. (**B**) The occupancy plots of each experimental group showing the duration in each area of novel tank.

**Figure 3:**
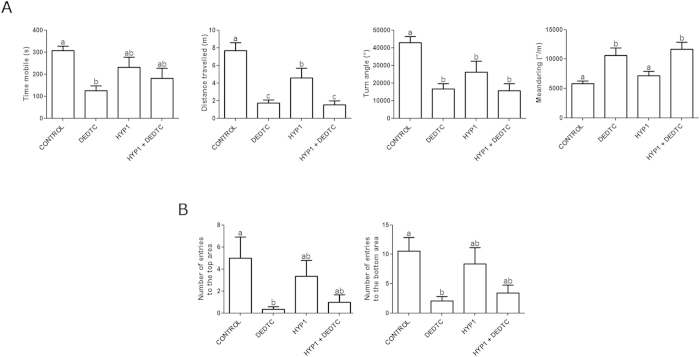
Locomotor activity and vertical exploration after hypoxia followed by normoxic treatment, with or without DEDTC. (**A**) Analysis of basic locomotor paramaters, such as time mobile, distance travelled, turn angle, and meandering. (**B**) Exploration of fish represented as transitions to top and bottom areas of the novel tank. Data represent means ± SEM (*n* = 9–12 per group). Different letters indicate statistical differences between groups at *p* < 0.05 level (two-way ANOVA followed by Tukey’s post hoc test).

The behavioral profiles of each group (*n* = 9–12 per group) are depicted as representative track plots (Fig. 2A). The animals kept on normoxia (NOR1) presented normal exploratory activity in all parts of the test tank during the trial. The individual swimming traces showed a marked reduction of exploratory activity in animals subjected to hypoxia (HYP1). However, fish subjected to hypoxia followed by exposure to DEDTC (HYP1 + DEDTC) exhibited a more intense decline in exploratory activity, a similar result observed in animals treated only with DEDTC. The occupancy plots (Fig. 2B) revealed a similar spatio-temporal exploration among groups, but DEDTC-treated groups showed a trend to decrease the swimming at the upper half of the apparatus. Two-way ANOVA indicated a significant effect of hypoxia on distance travelled (*F*[3, 39] = 4.576; *p* = 0.038) and turn angle (*F*[3, 39] = 4.126; *p* = 0.049) (Fig. 3A). Contrastingly, DEDTC significantly altered locomotion-related parameters, as time mobile (*F*[3, 39] = 11.286; *p* = 0.002), distance travelled (*F*[3, 39] = 33.898; *p* < 0.001), turn angle (*F*[3, 39] = 18.266; *p* < 0.001), and meandering (*F*[3, 39] = 21.623; *p* < 0.001). Post-hoc comparisons showed that although both HYP1 and HYP1 + DEDTC groups did not present significant changes in time mobile (Fig. 3A), DEDTC exposure per se caused a decrease in this parameter. The animals subjected to hypoxia (HYP1) also presented a markedly reduction in the distance travelled and turn angle (Fig. 3A). The administration of DEDTC after hypoxia episode (HYP1 + DEDTC) did not reverse these effects and also decreased the distance travelled, as observed in the DEDTC group. In addition, the hypoxia episode (HYP1) caused no changes on meandering (Fig. 3A), while both DEDTC-treated groups (DEDTC and HYP1 + DEDTC) increased the respective parameter.

The analysis of the vertical exploration showed that the time spent in bottom and top areas did not significantly change (data not shown). Furthermore, the transitions to the top and bottom areas (Fig. 3B) were unaltered in both HYP1 and HYP1 + DEDTC groups, while fish treated with DEDTC alone had a significant decrease in the number of transitions to both areas.

### Mitochondrial Activities in the Brain

**Figure 4:**
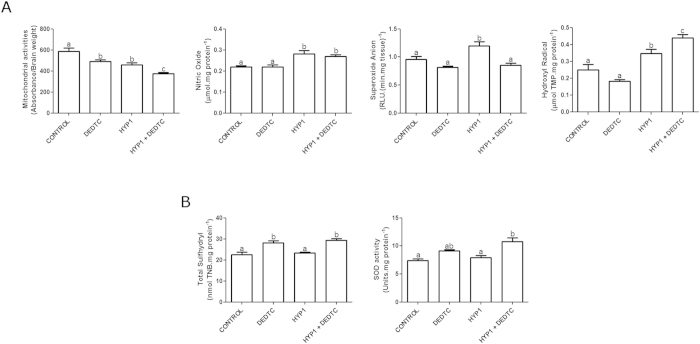
Brain effect of hypoxia followed by normoxia, with or without DEDTC, on mitochondrial dehydrogenase activities, levels of reactive species and the antioxidant-related responses. (**A**) Mitochondrial activities (*n* = 7–9 per group), levels of NO•, O_2_•^−^ and •OH scavenger capacities (*n* = 4–5 per group). (**B**) SH content and SOD activity (*n* = 4–5 per group). Data are presented as the means ± SEM. Distinct letters indicate statistical differences between groups at *p* < 0.05 level (two-way ANOVA followed by Tukey’s post hoc test).

The statistical analyses indicated a significant effect of hypoxia (*F*[3, 27] = 33.423; *p* < 0.001) and DEDTC (*F*[3, 27] = 17.901; *p* < 0.001) on the activity of the mitochondrial dehydrogenases (*n* = 7–9 per group). In fact, animals subjected to hypoxia (HYP1) had decreased mitochondrial activities (Fig. 4A) and fish treated with DEDTC after hypoxia (HYP1 + DEDTC) did further decrease this parameter. The DEDTC group also showed a significant decline in mitochondrial dehydrogenase activities.

### Cerebral Reactive Species

Two-way ANOVA showed a significant effect of hypoxia on the reactive species, like nitric oxide (NO•) (*F*[3, 12] = 24.121; *p* < 0.001), superoxide anion (O_2_•^−^) (*F*[3, 13] = 5.880; *p* = 0.031), and hydroxyl scavenger capacity (•OH) (*F*[3, 13] = 56.502; *p* < 0.001) (*n* = 4–5 per group). The DEDTC also presented substantial effects on the levels of O_2_•^−^ (*F*[3, 13] = 17.617; *p* = 0.001), while a significant interaction of hypoxia and DEDTC treatment on •OH scavenger capacity was observed (*F*[3, 13] = 11.239; *p* = 0.005). The comparison among groups showed that NO• levels (Fig. 4A) increased in animals subjected to hypoxia (HYP1) and the treatment with DEDTC after hypoxia (HYP1 + DEDTC) did not reverse this effect. Hypoxic episode (HYP1) increased O_2_•^−^ levels (Fig. 4A), while the fish subjected to hypoxia followed by DEDTC treatment (HYP1 + DEDTC) showed similar O_2_•^−^ content in comparison to control group. The measure of •OH scavenger capacity (Fig. 4A) on each group demonstrated that animals had an elevated content of •OH after hypoxia (HYP1), but treatment with DEDTC in animals subjected to hypoxia (HYP1 + DEDTC) did further increase this effect.

### Brain Antioxidant Responses

The statistical evaluation of antioxidant responses indicated a significant effect of DEDTC on sulfhydryl content (SH) (*F*[3, 14] = 53.519; *p* < 0.001) (*n* = 4–5 per group). The post hoc analyses showed that animals subjected to hypoxia did not alter SH content (HYP1), while DEDTC exposure after hypoxia caused an increase in the thiol levels (HYP1 + DEDTC) (Fig. 4B). Also, the SH content was substantially elevated in animals only treated with DEDTC. Two-way ANOVA analysis showed a significant effect of DEDTC (*F*[3, 12] = 23.644; *p* < 0.001) and hypoxia (*F*[3, 12] = 5.433; *p* = 0.038) on superoxide dismutase (SOD) activity (*n* = 4–5 per group). The post hoc analyses indicated that hypoxia caused no change in SOD activity (HYP1) (Fig. 4B), but treatment with DEDTC in zebrafish subjected to hypoxia resulted in a significant increase on enzyme activity.

## Discussion

The investigation of new potential drugs that prevent cerebral dysfunctions related to O_2_ deprivation is stimulated due to the elevated number of people affected worldwide. Specifically, the participation of the chelatable Zn in the pathophysiology of hypoxia/ischemia has attracted the attention of many researchers. During the ischemic and hypoxic episodes, the increased levels of chelatable Zn may induce neurotoxic events, culminating in brain damage^4^,^5^. Therefore, chemical agents with Zn chelating properties have been considered potential candidates as preventive compounds. Here, although DEDTC has exerted chelation of Zn content in the brain, our investigation on hypoxia model of zebrafish demonstrated harmful effects of DEDTC on exploratory behavior and oxidative status (Table 1).

In accordance with previous data^15^, we showed that hypoxia increased the levels of chelatable Zn in the PGz area of zebrafish brain. Furthermore, DEDTC exposure decreased the content of chelatable Zn close to the control levels, as previously described in brain hypoxia and ischemic stroke models after administration of Zn chelator^5^,^16^. Importantly, Zn chelators could decrease the levels of Zn indirectly due to the inhibitory effect on chelatable Zn release in CNS. It has been reported that the levels of chelatable Zn are mainly elevated by NO•, a molecule widely produced during ischemic events^17^ and hypoxia^5^. Based in our results, we suggest that DEDTC acts by direct Zn chelation in zebrafish brain since NO• levels remained unchanged after DEDTC treatment.

Specifically, the PGz is a cerebral area of optic tectum with abundant and well-located content of chelatable Zn^7^, being related to visual functions of zebrafish. In fact, chelatable Zn has been identified as important neuromodulator in visual pathway of this species^18^. Because of the intrinsic functions of PGz on visual skills, which play a key role in the exploratory profile of zebrafish (e.g. visual acuity), we sought to investigate whether the attenuating effect of DEDTC on the content of chelatable Zn in PGz is associated to behavioral activity. Using the novel tank test, we confirmed an exploratory deficit caused by hypoxia in zebrafish^14^, indicating that chelatable Zn content in PGz may be an important modulator of these behaviors. It is conceivable that the partial chelation of Zn by DEDTC did further exacerbate the exploratory impairments caused by hypoxia, but this effect was similar to that observed after DEDTC treatment alone, which did not alter Zn levels in PGz. Therefore, we postulate that hypoxia and DEDTC exposure probably modulate distinct mechanisms involved on exploratory deficit of zebrafish: one associated to increased Zn content in PGz (played by hypoxia) and another non-related to Zn content of PGz (played by DEDTC).

In this context, we verified whether oxidative stress could have a role on exploratory impairment caused by hypoxia and mainly DEDTC. After 1 h of hypoxia episode, fish exhibited a decrease on mitochondrial activities and a significant increase in all reactive species assessed, suggesting that mitochondrial damages can have led to increased levels of reactive species, such as observed in stroke and other hypoxia models^19^,^20^. Importantly, these results indicate that both reactive species and exacerbated Zn content in PGz may be involved in exploratory deficits induced by hypoxia. Interestingly, the fish exposed to hypoxia and DEDTC showed an attenuating effect on O_2_•^−^ levels, did not show significant changes in NO• levels, but presented exacerbated mitochondrial damage, which possibly contributed to the substantial decrease of •OH scavenger capacity. All these findings indicate that these fish presented a more critical exploratory deficit due to cumulative effects of hypoxia and DEDTC on oxidative stress. These data reinforce the assumption that DEDTC may have triggered a pro-oxidant effect in this study. Nevertheless, there are two events that contrast to this hypothesis. Firstly, we observed a lower rather than higher O_2_•^−^ levels in animals exposed to hypoxia and DEDTC. However, as O_2_•^−^ is a substrate for the production of •OH in Haber-Weiss reactions^21^, these effects could occur simultaneously in animals treated with DEDTC after hypoxia, supporting the pro-oxidant action of DEDTC. Finally, DEDTC alone could undermine this hypothesis, because mitochondrial damage was not associated to changes in reactive species levels in this experimental group.

Considering that an induction of protective molecules could be responsible for maintaining the normal levels of reactive species in the presence of DEDTC, we investigated the levels of some cellular antioxidants. Even with higher levels of reactive species, the animals showed no changes on the antioxidant parameters 1 h after the hypoxic episode. It has been reported that the increased activities of antioxidant enzymes (e.g., SOD) in the brain occur as a late event in ischemia and hypoxia^22^,^23^. We have previously observed that the mitochondrial damage generated by hypoxia in the brain of zebrafish is reverted after 48 h, indicating a possible induction of antioxidant defenses on this time^14^. Therefore, our results show that 1 h after hypoxia was ineffective to stimulate these antioxidant responses, which certainly contributed for exploratory deficit observed. In contrast, the administration of DEDTC increased SOD activity and sulfhydryl content in the brain of animals subjected to hypoxia. Apparently, these results support the existence of a balance between reactive species and antioxidants, because these cellular defenses were induced in fish with elevate mitochondrial damage and •OH content. If both mechanisms are working together, it is possible to conclude that antioxidants were insufficient to protect of intense exploratory deficit promoted by DEDTC treatment in HYP1 + DEDTC. This fact suggests that even attenuating the increased content of chelatable Zn, DEDTC probably played a pro-oxidant effect. In accordance with this data, Rahden-Staroń *et al.*^12^ have shown that the administration of DEDTC on cell culture generates oxidative damage in proteins and lipids along with the induction of antioxidant enzymes and production of glutathione. In fact, we verified that DEDTC *per se* promoted a significant increase of sulfhydryl content as a probable response to toxic mechanisms, since we observed mitochondrial damage and exploratory deficit in these same animals. Thus, our data reinforce the hypothesis that DEDTC presented a pro-oxidant effect in the brain of animals subjected to hypoxia.

Although our results involve at least one putative mechanism by which DEDTC did not reverse the behavioral effects induced by hypoxia, there are some points to be considered in this work. Firstly, we could postulate a protective effect of DEDTC in other concentrations. Indeed, we tested a concentration-response of DEDTC in our previous study^24^, where the higher concentrations of DEDTC (1 and 5 mM) decreased overly the chelatable Zn content in the brain of zebrafish, leading to gross behavioral impairments (e.g. seizure-like behavior). In contrast, 0.2 mM of DEDTC caused smaller effects on zebrafish, representing the highest concentration of DEDTC with low cerebral side effects.

Another point to be considered is the opposite effect of DEDTC described in our study in comparison to previous findings^15^. Using 0.25 mM DEDTC Yu and Li^16^ reported an enhanced tolerance to hypoxia associated to a neuroprotective action of the compound on chelatable Zn content and mitochondrial activities of zebrafish. We hypothesize that these divergent data could be attributed to distinct experimental approaches. For example, in the previous work, the mitochondrial activities and chelatable Zn content were evaluated in other recovery periods (24 h and 30 min, respectively) and after a DEDTC treatment during highly lethal hypoxic condition (dissolved O_2_ less than 0.8 mg/L). Here, we investigate all parameters associated to behavioral task and oxidative stress under a post-hypoxic treatment of DEDTC. Because the underlying relevance for the treatment of disorders associated with hypoxia, we adopt the post-hypoxia treatment of DEDTC, which probably determined the results presented herein.

In summary, our results showed that hypoxia resulted in abnormally high Zn accumulation in the brain. Additionally, DEDTC mitigated the increased Zn content caused by hypoxia, but this effect did further increase the behavioral impairments. In an analysis focused on oxidative stress parameters, we reinforced the idea that DEDTC performed a pro-oxidant action in animals subjected to hypoxia, which was probably involved in the exploratory deficit observed. Therefore, based on a new hypoxia model, our data do not support DEDTC use for neuroprotection in dysfunctions involving hypoxia.

## Methods

### Animals

A total of 160 male and female (50 : 50 ratio) wild-type adult zebrafish (5–7 months-old) were obtained from a commercial local distributor (Delphis, RS, Brazil). The animals were kept in aquarium rack system (Zebtec, Tecniplast, Italy) under density of 5 fish/L and light/dark cycle of 14/10 h (lights on at 7:00 am) for at least 2 weeks prior to the experiments. Fish were fed twice daily with commercial flake fish food (alcon BASIC^®^, Alcon, Brazil) and once a day with *Arthemia sp*. The experiments were performed using reverse osmosis water supplemented with 0.018 g.L^−1^ Instant Ocean^®^ salt. At these conditions, normoxic water presented 7.5 mg.L^−1^ O_2_, 500 μS/cm^2^ of conductivity and pH 7.2. To ensure an adequate aeration in the tanks, the O_2_ dissolved in water was constantly measured with an oximeter (Instrutherm, SãoPaulo, SP, Brazil). All experiments were in accordance with National Institute of Health Guide for Care and Use of Laboratory Animals. Experimental protocols were approved by Ethics Committee of Universidade Federal do Rio Grande do Sul (number 24471 – CEUA).

### Hypoxia Model in Zebrafish

The hypoxia model in zebrafish was performed based in the methods previously described^13^,^14^. Initially, pure N_2_ was perfused into the water until the dissolved O_2_ reach 1.5–1.7 mg.L^−1^. Next, two fish were transferred to the hypoxia chamber and the apparatus was hermetically sealed in order to provide a closed air tight system. The O_2_ levels (1.5–1.7 mg.L^−1^) in the water were continuously monitored by an oximeter. Importantly, each hypoxia trial consisted in keeping the fish at low O_2_ levels up to reach the third stage of the hypoxia (maintenance of opercular beats with brief movements), characterized by a critical but non-lethal condition^14^.

### Treatment Conditions and Experimental Groups

After hypoxia, each animal was individually kept in tanks containing 400 mL of normoxic water (7.5 mg.L^−1^ O_2_), in absence or presence of 0.2 mM DEDTC for 1 h. This DEDTC concentration was chosen due to low cerebral side effects^24^ and potential neuroprotective action in zebrafish^15^. Furthermore, the 1 h period was chosen because zebrafish still present behavioral and neurochemical changes caused by hypoxia^14^. Animals were separated in four groups: CONTROL, animals kept under normoxia for 1 h; DEDTC, animals kept under normoxia and DEDTC for 1 h; HYP1, animals subjected to hypoxia and kept under normoxia for 1 h; and HYP1 + DEDTC, animals subjected to hypoxia and kept under normoxia and DEDTC for 1 h. Importantly, the normoxic levels of O_2_ in the water were kept constant during treatments (7.5 mg.L^−1^ O_2_). After treatments, fish were anesthetized using ice-cold water and euthanatized by decapitation to remove the brain.

### Histochemical Quantification of Chelatable Zn in the Brain

The chelatable Zn content from PGz was histochemically stained by Neo-Timm method, as performed in zebrafish brain^7^. The PGz was evaluated due to its abundant content of chelatable Zn and its intrinsic relation with optic tectum, which plays a role in zebrafish visual processing. Briefly, brains were immediately immersed in 3% glutaraldehyde containing 0.1 M phosphate buffer (pH 7.4) for 24 h, and then transferred to sodium sulfide solution (1% Na_2_S) containing 0.12 M Millonig’s buffer for 24 h. Slices (30-μm-thick) were produced in vibroslicer (VTS-1000;Leica), and mounted on slides. The histochemical staining was performed by incubation of the slices in a solution containing 120 mL of 50% gum Arabic, 30 mL of hydroquinone (1.85 g), 20 mL of citric acid (5.12 g) plus sodium citrate (4.72 g), and 1 mL of silver nitrate (0.17 g) for 120 min. The reaction was performed in a dark room, and only ultrapure and metal-free solutions were used. The NIS Elements AR 2.30 software (Nikon) was used to capture the images from a light microscope (Nikon Eclipse E-600) coupled with a camera (Nikon DXM1200C CCD). The optical density of the total area of the PGz of zebrafish was determined to quantify chelatable Zn stained by Neo-Timm under 10x magnification using ImageJ 1.37v software. The results were expressed in arbitrary units (a.u.) followed by a normalization in relation to the control.

### Novel Tank Test

After treatments, the behavioral activity of some fish was evaluated by the novel tank as previously described^25^. The apparatus consisted in a plastic tank (23.9 cm along the bottom x 28.9 cm at the top x 15.1 cm high) virtually divided into three horizontal areas (bottom, middle, and top) and with five sections per area. The tank was filled with 1.5 L of normoxic water and placed on a stable surface with all environmental interferences kept to a minimum. Animals were individually transferred to the novel tank and their behavior was recorded for 6 min. The swimming activity was acquired by a webcam (Microsoft^®^ LifeCam 1.1 with Auto-Focus) and all behavioral parameters were measured by video tracking software at 30 frames/s (ANY-maze^®^, Stoelting CO, USA). The locomotor activity was assessed by measuring the total time mobile, distance travelled, turn angle (the variations in direction of the center point of the animal), and meandering (turn angle divided by distance travelled). Since the zebrafish tend to gradually explore upper areas in a novel apparatus^26^, the vertical exploration was also evaluated by measuring the number of transitions in top and bottom areas. All tests were performed during the same time frame each day (10:00 am - 4:00 pm), and the water was replaced every session.

### Measurement of the Mitochondrial Activities in the Brain

The activities of brain mitochondrial dehydrogenases were evaluated by the level of formazan produced from 2,3,5-triphenyltetrazolium chloride (TTC)^14^. The whole brains were incubated in 1 mL of phosphate buffer solution (PBS, pH 7.4) containing 2% TTC (Sigma–Aldrich, St. Louis, MO, USA) at 37 °C for 40 min in dark room. Next, TTC was removed and 10% formalin dissolved in PBS (pH 7.4) was added in order to terminate the reaction. The brains were dried at 40 °C for 2 h and separately weighed in an analytical balance. Then, each brain was incubated with 200 μL of dimethylsulfoxide (DMSO) (Sigma–Aldrich, St. Louis, MO, USA) in 96 wells plates protected from light. The plates were kept under constant agitation for 4 h to solubilize the formazan produced from TTC. The pink-to-red eluate of formazan was read at 490 nm in a microplate reader and the data were expressed in absorbance per tissue dry weight (g).

### Oxidative Status in the Brain

Four brains were pooled (1*n* = 4 brains) to measure NO•, O_2_•^−^, •OH, SH, and SOD activity. Due to the unequal amount of samples needed to perform each technique, the use of pooled brains allowed the analysis of all oxidative stress parameters by using a smaller number of animals. The samples were immediately kept on ice and were freshly homogenized (1:20, w/v) in 20 mM sodium phosphate buffer, pH 7.4 containing 140 mM KCl. The homogenates were centrifuged at 800 g for 10 min at 4 °C to separate nuclei and cell debris. Protein content was measured according to Lowry *et al.*^27^ using bovine albumin as standard. The analyses of oxidative stress parameters and protein content were run in duplicate.

### Evaluation of Reactive Species

The O_2_•^−^ levels were detected by measuring the chemiluminescence of lucigenin based in previous works^28^,^29^. The supernatants (50 μL) were added in medium reaction (100 μL) with Krebs HEPES buffer in the presence of lucigenin (20 μmol/L). Since hypoxia induces an increase in O_2_•^−^, the maximum acceptable concentration of lucigenin was used in order to increase sensitivity of the detection of O_2_•^−^ without artificial self-generation of O_2_•^−^ in the samples^30^. The luminescence of samples was evaluated in a luminescence counter (Perkin Elmer, MicroBeta TriLux) for 10 min. Values of blank chambers containing the same reagents were recorded as background, which were subtracted from those from their corresponding samples. The data were normalized to tissue weight and expressed in RLU (relative luminescence unit)/min/mg tissue.

Nitric oxide content was assessed from Griess reaction using a protocol based on Green *et al.*^31^. The incubation was performed in 96-well plates containing 50 μL of supernatant and 50 μL of Griess reagent (1:1 mixture of 1% sulfanilamide in 5% phosphoric acid and 0.1% dihydrochloride naphthylethylenediamine in water). A standard curve of NO• was obtained with known concentrations of nitrite (1.5–100 μM). Plates were protected from light and kept at room temperature for 10 min. The absorbance was measured at 543 nm and nitrite concentration was represented as μmol of nitrite/mg protein.

The deoxyribose assay was used to evaluate the •OH production from the auto-reduction of ferric citrate complex^32^. Briefly, the pentose is attacked by •OH with releasing thiobarbituric acid (TBA) reactive substances. The samples (25 μL of supernatant) were incubated at 37 °C for 1 h in dark room with a medium containing 3 mM 2-deoxy-D-ribose, 20 μM FeCl_3_, 100 μM EDTA, 500 μM H_2_O_2_ and 100 μM ascorbate. Afterwards, 10% TCA and 0.67% TBA were added and incubated for 1 h in boiling water bath. The absorbance was determined at 532 nm and 1,1,3,3-tetra-methoxypropane (TMP) was used as standard. The results were expressed as μmol TMP/mg protein.

### Antioxidant Analysis

The assay of sulfhydryl content was based on the reduction of 5,5′-dithio-bis(2-nitrobenzoic acid) (DTNB) by thiols^33^. Briefly, 14 μL of homogenate were added to 270 μL of PBS (0.1 M, pH 7.4) containing 1 mM EDTA. Then, 16 μL of PBS containing 10 mM DTNB were added. Controls without DTNB or samples were conducted simultaneously. Subsequently, a 30-min incubation was performed at room temperature in a dark room. The absorption was measured at 412 nm and the results were presented as nmol TNB/mg protein.

The superoxide dismutase activity was analyzed by autoxidation capacity of pyrogallol as obtained in Marklund method^34^. The inhibition of pyrogallol autoxidation occurs by SOD activity, which is determined in a medium containing 15 μL of sample and 235 μL of a solution of 50 mM Tris-HCl (pH 8.2), 1 mM EDTA, 30 μM catalase, and 24 mM pyrogallol. The product was read at 420 nm. The inhibition of pyrogallol autoxidation (50%) was defined as one unit of SOD and the specific activity was expressed as units/mg protein.

### Statistical analysis

The values were expressed as means ± SEM. The behavioral and neurochemical parameters were analyzed by two-way ANOVA, using hypoxia and DEDTC treatment as factors. Post hoc comparisons were performed by Tukey’s test. Statistical significance was set at *p* < 0.05 level.

## Additional Information

**How to cite this article**: Braga. M. M. *et al.* Brain zinc chelation by diethyldithiocarbamate increased the behavioral and mitochondrial damages in zebrafish subjected to hypoxia. *Sci. Rep.* **6**, 20279; doi: 10.1038/srep20279 (2016).

## Figures and Tables

**Table 1:** Behavioral and neurochemical effects on each experimental group in comparison to control.

**TYPE**	**PARAMETER**	**DEDTC**	**HYP1**	**HYP1 + DEDTC**
BEHAVIORAL	Time mobile	—	0	0
	Distance Travelled	−−	—	−−
	Turn angle	—	—	—
	Meandering	+	0	+
	Entries to top area	—	0	0
	Entries to bottom area	—	0	0
NEUROCHEMICAL	Chelatable Zn	0	+	0
	Mitochondrial activities	—	—	—
	Nitric oxide	0	+	+
	Superoxide anion	0	+	0
	Scavenger capacity (•OH)	0	+	++
	Total Sulphydryl	+	0	+
	SOD activity	0	0	+

DEDTC: animals kept under normoxia and DEDTC for 1 h; HYP1: animals subjected to hypoxia and kept under normoxia for 1 h; HYP1 + DEDTC: animals subjected to hypoxia and kept under normoxia and DEDTC for 1 h.

(0), no change in relation to control; (−), reduction in relation to control; ( + ) elevation in relation to control.
